# Pharmacokinetics Study of Herb–Drug Interaction of *Berberine* and Glipizide in Beagle Dogs Using UPLC-MS/MS

**DOI:** 10.1155/ianc/7941435

**Published:** 2025-07-28

**Authors:** He Qi, Wenjiong Wang, Xianghan Zhang, Bingyang Shang

**Affiliations:** Innovation Practice Platform for College Student, College of Basic Medicine and Forensic Medicine, Henan University of Science and Technology, Luoyang, Henan, China

**Keywords:** *berberine*, glipizide, HDIs, pharmacokinetics, UPLC-MS/MS

## Abstract

Based on the establishment and validation of a UPLC-MS/MS method for detecting glipizide in beagle plasma, the herb–drug interaction (HDI) between berberine and glipizide was studied. After gradient elution separation of glipizide and internal standard, multiple reaction monitoring was used for detection in positive ion mode. The ion reactions used for quantitative analysis were glipizide m/z 446.0 ⟶ 321.0 and IS m/z 307.1 ⟶ 220.0. Six beagle dogs were treated with glipizide alone and berberine intervention, and the pharmacokinetic changes of glipizide were compared. The UPLC-MS/MS method has good linearity and the advantages of being green, simple, sensitive, and fast. After continuous administration of berberine to beagle dogs for 7 days, the pharmacokinetic process of glipizide changed with *C*_max_, AUC_(0-t)_ and AUC_(0-∞)_ increasing, t_1/2_ prolonging, and CL and Vd decreasing. When using combination therapy, attention should be paid to possible HDI.

## 1. Introduction

Diabetes mellitus (DM) is an endocrine disorder, which is mainly caused by pancreatic islets β cellular dysfunction or destruction leading to relative or absolute insulin deficiency. Diabetes is one of the challenges facing global health, and it is growing fastest, bringing huge burden to the affected individuals and their families [1]. In the past few decades, the prevalence of DM has increased significantly, mainly due to the continuous rise in the incidence rate of Type 2 DM [2]. The incidence rate of diabetes is increasing rapidly, which usually leads to serious metabolic diseases and serious complications [3]. Although antidiabetes drugs and insulin have been used to treat DM, there is still no fundamental cure for DM [1].

Glipizide is a second-generation sulfonylurea drug used to treat Type 2 diabetes, which stimulates the pancreas β cells secrete insulin and takes action [4]. After oral administration, glipizide is completely absorbed from the gastrointestinal tract and cleared through extensive metabolism in the liver. It is mainly metabolized into inactive metabolites through genetic polymorphism of CYP2C9 enzyme [5].

Berberine (BBR) is an isoquinoline alkaloid widely distributed in plants, such as *Coptis chinensis*. The bioavailability of BBR is low, but it can interact with the intestinal microbiota and affect various diseases, such as DM, hyperlipidemia, atherosclerosis, liver disease, intestinal diseases, mental disorders, and autoimmune diseases [6]. BBR has antidiabetes activity and can reduce blood glucose levels, increase insulin secretion, and weaken glucose tolerance and insulin resistance by activating the AMPK pathway. Additionally, BBR also shows various other activities, such as antifat, antihyperlipidemia, anti-inflammatory, and antioxidant [7]. BBR regulates PI3K/AKT/NFκB and MAPK pathways, which improve inflammation and cell apoptosis in dry eye syndrome, and may become candidate drugs for the treatment of dry eye syndrome [8]. In addition, BBR also has neuroprotective effects, and the molecular mechanisms responsible for BBR neuroprotection include reducing oxidative stress, alleviating inflammation, inhibiting cell apoptosis pathways, promoting autophagy processes, and regulating CYP450 enzyme activity, neurotransmitter levels, and gut microbiota composition [9].

Complementary and alternative drugs (CAM) extracted from natural products or plant–based substances are increasingly being used for the treatment of diseases. DM is a disease that has a good response to CAM treatment, especially plant natural products. About 78% of DM patients use herbs and supplements as alternative treatment [10]. Clinically, when taking glipizide, patients with DM will also take CAM for auxiliary treatment, such as BBR.

The components of herbal compounds can cause herb–drug interactions (HDIs) as they can act as reversible inhibitors, irreversible inhibitors, or inducers of CYP450 enzymes or have synergistic, additive, or antagonistic effects on the pharmacological activity of drugs [11]. Because glipizide is metabolized through CYP2C9, BBR can regulate the activity of CYP450. Therefore, when BBR is combined with glipizide, HDIs between BBR and glipizide will occur. For this reason, by establishing a green, simple, sensitive, and rapid UPLC-MS/MS method for measuring the concentration of glipizide in beagle dog plasma, the HDIs between BBR and glipizide were explored based on pharmacokinetics.

## 2. Materials and Methods

### 2.1. Reagents and Drugs

Methanol and acetonitrile were purchased from Tianjin Kemiou Chemical Reagent Co. and were of first-level chromatographic purity. Ultrapure water after filtration by Milli-Q reagent system (Millipore, Bedford, USA) was prepared. The National Institute for Food and Drug Control has provided reference standards for glipizide (100281–201604) and fluconazole (130695–201501). BBR hydrochloride tablets (each table contains 100 mg of BBR hydrochloride) were produced by Jiangsu Yabang Epson Pharmaceutical Co., Ltd.

### 2.2. Solution Preparation

Glipizide was weighed 10 mg and volume fixed in 10 mL volumetric flasks to obtain 1 mg/mL of standard stock solution, which were then diluted gradually with HPLC grade methanol to obtain glipizide working solution at concentrations of 100, 10, and 1 μg/mL, respectively. The plasma standard curve of glipizide (10, 25, 50, 100, 250, 500, 1000, and 2000 ng/mL) was obtained by adding standard application solutions of different concentrations and volumes to blank plasma, and QC samples (25, 500, and 1500 ng/mL) were prepared using the same method.

The fluconazole stock solution of 1 mg/mL was obtained by accurately weighing 10 mg of fluconazole, dissolving it in methanol, and diluting it to 10 mL. A certain amount of stock solution was taken and diluted with acetonitrile to form 150 ng/mL of internal standard application solution.

### 2.3. Apparatus

The ultrahigh liquid chromatograph was part of the Waters Acquity series, which included the quaternary solvent manager (1860–15018), the automatic injection manager (1860–15017), and workstation, as well as Waters products. The mass spectrometer was Xevo TQ-S triple quadrupole mass spectrometer, a product of Waters company.

### 2.4. Chromatographic and Mass Spectrometry Conditions

The chromatographic column was an Equity BEH C18 column (2.1 × 50 mm, 1.7 μm). The mobile phase was acetonitrile 0.1% formic acid system, and the gradient elution procedure was as follows: the proportion of acetonitrile was kept at 10% for 0–0.5 min, the proportion of acetonitrile increased from 10% to 90% for 0.5–1.0 min, the proportion of acetonitrile in the mobile phase was 90% for 1.0–1.4 min, and the proportion of acetonitrile in the mobile phase continuously decreased from 90% to 10% for 1.4–2.0 min. The flow rate was set at 0.40 mL/min, the column temperature was set at 40°C, and the sample room temperature was set at 4°C.

The ion source was an electric spray ion source, which was detected by a positive ion method. Glipizide and IS fluconazole were detected by multiple reaction monitoring (MRM) method. The parent ion and daughter ion used for quantitative analysis were glipizide m/z 446.0 ⟶ 321.0 and IS m/z 307.1 ⟶ 220.0, respectively. The time of dwell, the voltage of cone, and the voltage of collision were 0.16 s, 30 V, and 20 V for glipizide, respectively. The time of dwell, the voltage of cone, and the voltage of collision were 0.16 s, 20 V, and 20 V for IS fluconazole, respectively. The MassLynx 4.1 software was used to acquire the parameters and data of the MS/MS system.

### 2.5. Processing Methods for Plasma Sample

In a 1.5-mL EP tube, accurately add 100 μL of the plasma sample to be tested and 200 μL of the internal standard working solution in sequence. After 1.0 min of vortex mixing, the mixture was centrifuged at 12000 rpm for 10 min. Then, the supernatant was placed into a sample bottle, and 2 μL of the solution was set for detection.

### 2.6. Methodological Validation

According to the “guiding principles for the validation of quantitative analysis methods for biological samples” in the Chinese Pharmacopoeia (2020 edition), the UPLC-MS/MS method was validated including specificity, standard curve, lower limit of quantification (LLOQ), accuracy and precision, stability, and matrix effect.

#### 2.6.1. Selectivity

This analysis method should be able to target the endogenous components of the analyte and plasma or other components in the sample. Suitable blank plasma from at least six subjects should be used to demonstrate selectivity.

#### 2.6.2. Standard Curve and LLOQ

The plasma standard solutions of glipizide with concentrations of 10, 25, 50, 100, 250, 500, 1000, and 2000 ng/mL were prepared, and processed according to the plasma sample processing method. After UPLC-MS/MS detection, the peak area As of glipizide and the peak area Ai of the internal standard were recorded. The standard curve was plotted with As/Ai as the *y*-axis and the corresponding concentration *x* as the *x*-axis. The minimum concentration of the standard curve was the LLOQ.

#### 2.6.3. Precision and Accuracy

The precision of an analytical method describes the degree of closeness to repeated measurements of the analyte, defined as the relative standard deviation (RSD %) of the measured value. The accuracy of an analytical method is the degree to which the measured value of the method is close to the indicated concentration of the analyte, expressed as (measured value/true value) ∗ 100%. The precision and accuracy of LLOQ and QC samples (25, 500, and 1500 ng/mL) were evaluated.

#### 2.6.4. Matrix Effects and Recovery

At least six batches of blank matrices from different subjects should be used to investigate matrix effects. By calculating the ratio of the peak area in the presence of matrix to the corresponding peak area without matrix, the matrix effect of each analyte and internal standard was calculated. The extraction recovery rate of the analyte is represented by dividing the peak area of the target analyte extracted from the biological sample matrix by the peak area generated by the standard sample. The matrix effects and recovery of QC samples (25, 500, and 1500 ng/mL) were evaluated.

#### 2.6.5. Stability

Stability must be ensured at every step of the analysis method. Stability tests should usually be conducted under the following conditions: room temperature storage, three freeze-thaw cycles, long-term freezing storage, and stability of the sample after treatment. The stability of QC samples (25, 500, and 1500 ng/mL) were evaluated.

### 2.7. Animal Experiments

Hubei Yizhicheng Biotechnology Co., Ltd. (Yingcheng, Hubei) provided the six beagle dogs (weight 8.5∼9.5 kg) with SCXK (Hubei) 2021-0020. The six beagle dogs were raised in the Animal Laboratory of Henan University of Science and Technology (Luoyang, China). The beagle dogs were housed in a room at 20°C–25°C, with a 12/12 h light/dark cycle, 40%–70% humidity, fed twice a day, and free water. All beagle dogs were prohibited from eating for 12 h before the experiment but were allowed to drink water freely. The animal laboratory of Henan University of Science and Technology approved the animal experiment plan (202307002).

HDI was evaluated by comparing the pharmacokinetics of glipizide alone with those of glipizide under BBR intervention. The dosage of BBR hydrochloride and glipizide in beagle dogs is calculated based on the human dose. Before taking glipizide, the blank blood was collected from every beagle dog as a control, then the six beagle dogs were orally administered with glipizide at a dose of 0.67 mg/kg, and the approximately 1.0 mL of venous blood was collected at the time point of 0.5, 1, 1.5, 2, 3, 4, 6, 9, 12, 16, and 24 h after administration. After a week of drug wash out period, the six beagle dogs were orally given BBR hydrochloride 10 mg/kg twice a day for seven consecutive days. Similarly, before taking glipizide, the blank blood was collected from every beagle dog as a control, then 0.67 mg/kg glipizide was orally administered to six beagle dogs, and approximately 1.0 mL of venous blood was collected at the time point of 0.5, 1, 1.5, 2, 3, 4, 6, 9, 12, 16, and 24 h after administration. The plasma was separated and frozen for testing.

### 2.8. Data Analysis

DAS 2.0 program was used to treat the plasma concentrations of glipizide alone administration and the plasma concentration of glipizide under BBR intervention. The statistical moment model was used to calculate the main pharmacokinetic parameters of glipizide. Paired *t*-test was used to compare the differences of pharmacokinetic parameters between glipizide alone and glipizide under BBR intervention.

## 3. Results

### 3.1. Method Validation

#### 3.1.1. Selectivity

**Figure 1:**
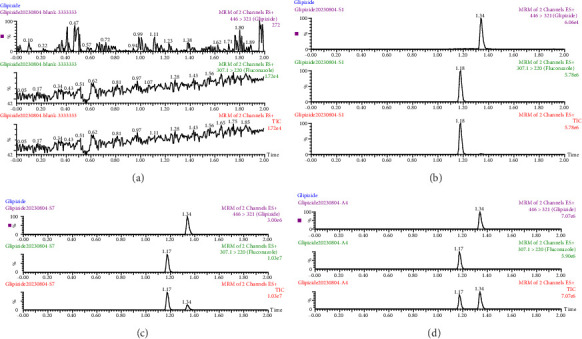
Typical chromatograms of glipizide and IS. (a) Blank plasma; (b) blank plasma containing glipizide (2 ng/mL) and IS; (c) blank plasma containing glipizide (1000 ng/mL) and IS; (d) a plasma following oral dosing with glipizide (561.19 ng/mL).

The selectivity of the UPLC-MS/MS has been verified through chromatograms of four samples: a beagle dog blank plasma, a blank plasma sample with the limit of quantification (LOQ) of the method, a plasma standard solution, and a plasma obtained from a beagle dog after glipizide administration. As illustrated in Figure 1, the peak shapes of glipizide and internal standard were good. The retention time of glipizide and internal standard was 1.34 and 1.17 min, correspondingly.

#### 3.1.2. LOQ and Standard Curve

The regression equation for the linear equation is *y* = 0.0033 *x* + 0.0178 (*r* = 0.999 3), with the ratio of the peak area of glipizide to the internal standard as the *y*-axis and the plasma concentration of glipizide as the *x*-axis. It indicated that glipizide was well linearity within the 10–2000 ng/mL range and the LLOQ was 10 ng/mL. The LOQ of the method was 2 ng/mL (Figure 1).

#### 3.1.3. Accuracy and Precision

Table 1 shows the results of precision and accuracy of glipizide at 10, 25, 500, and 1500 ng/mL. The precision (% RSD) did not exceed 7.86%. Accuracy (%) was around 100%.

#### 3.1.4. Matrix Effect and Recovery

At 25, 500, and 1500 ng/mL, the extraction recoveries of glipizide were (80.93 ± 4.32)%, (84.64 ± 4.05)%, and (83.45 ± 2.14)%, respectively. The matrix effect did not affect the detection of glipizide with the value of 96.40%∼102.86% (Table 1).

#### 3.1.5. Stability

The test specimens were examined and analyzed for stability at 25, 500, and 1500 ng/mL. It is seen in Table 2 that the results were stable under four different conditions, and the RSD values of glipizide were less than 10%.

### 3.2. The HDIs of BBR and Glipizide

**Figure 2:**
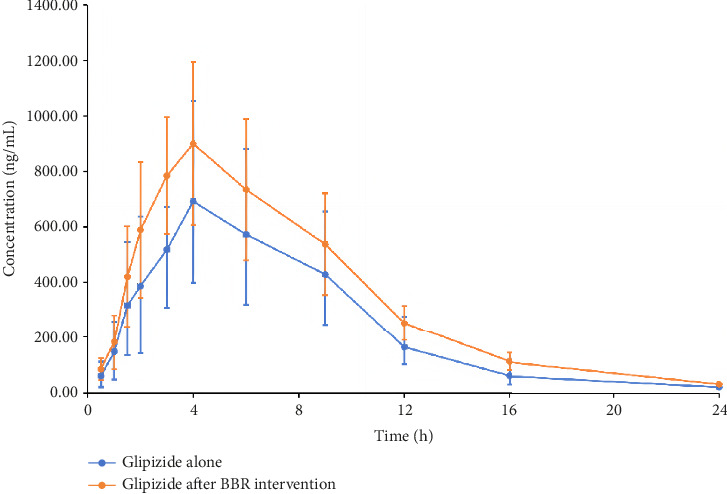
Mean drug concentration–time curves of glipizide in beagle dogs.

After continuous administration of BBR for 7 days, administration of glipizide resulted in a peak concentration (*C*_max_) 52.71% higher than when used alone, with AUC_(0-t)_ and AUC_(0-∞)_ being 60.29% and 61.66% higher, respectively. Meanwhile, after intervention with BBR, the t_1/2_ of glipizide was slightly prolonged, and CL and Vd were reduced. After intervention with BBR, there were significant differences in the main pharmacokinetic parameters (*C*_max_, AUC_(0-t)_, AUC_(0-∞)_, and CL) of glipizide compared to when used alone. BBR can increase the plasma exposure of glipizide. The average drug concentration time curves of glipizide alone and glipizide after BBR intervention are shown in Figure 2, and the main pharmacokinetic parameters of glipizide are shown in Table 3.

## 4. Discussion

### 4.1. Methodological Development and Optimization

There have been reports on the UPLC-MS/MS method for detecting the concentration of glipizide in human plasma [12]. In this study, the acetonitrile precipitation plasma protein method was still used, and a UPLC-MS/MS method for detecting glipizide in beagle plasma with fluconazole as the internal standard was established.

The chromatographic peak shapes and separation effects of the target compound were compared using methanol 0.1% formic acid aqueous solution, methanol water, and acetonitrile 0.1% formic acid aqueous system as mobile phases. The results showed that the target compound could be effectively separated under all three mobile phase systems, and the chromatographic peak shapes were good. Due to the PKa of 5.9 for glipizide and considering that adding an appropriate amount of acid in the mass spectrometry can provide an ionization effect, and due to the low mass transfer resistance of acetonitrile, acetonitrile-0.1% formic acid aqueous solution was ultimately selected as the mobile phase.

Using triple quadrupole mass spectrometry for quantitative and qualitative analysis, optimize mass spectrometry parameters for each target substance by injecting samples separately, mainly including the selection of qualitative and quantitative ion pairs, cone voltage, and collision energy. Due to the polarity of glipizide, ESI was used for ionization, and the flow rate and temperature of the atomizing gas and drying gas were optimized. Under ESI + conditions, the parent ion ([*M* + *H*]+) was determined by Q1 full scan, and characteristic fragment ions were selected by ion accumulation scan. Meanwhile, optimize the CE value to increase the abundance of fragment ions.

At present, protein precipitation is the most commonly used method for biological sample pretreatment. Common protein precipitants include methanol, acetonitrile, ethanol, acetone, perchloric acid, and tetrahydrofuran. Acetonitrile, as a protein precipitant, has a high protein precipitation rate and fewer impurities. Therefore, this study chose acetonitrile as the protein precipitant for blood sample pretreatment. From the perspective of precipitation efficiency, sufficient vortexing of acetonitrile with the sample for 1 min can cause precipitation of most proteins. In addition, the use of acetonitrile protein precipitation method greatly shortens the pretreatment time of blood samples, indicating that acetonitrile can be used as a good protein precipitant for pretreatment of glipizide blood concentration detection.

### 4.2. HDIs

Herbs have been used as food and medicine for a long time, often in combination with other prescription drugs. Although these herbs are considered natural and safe, many of them can interact with other drugs, causing potential dangerous side effects or reducing the benefits of the medication. The complex and diverse pharmacological effects of active ingredients in herbs inevitably alter the pharmacokinetics of chemical drugs administered in vivo [13]. Concurrently using herbs and traditional medicines is a potentially dangerous practice [14].

When taken together with prescription drugs, plant-based dietary supplements may cause clinically significant HDIs. Pharmacodynamic HDI describes the interactions between plant chemicals and traditional drugs at the drug receptor level, and pharmacokinetic HDI originates from the induction and/or inhibition of human drug metabolism enzymes and/or transporters mediated by plant chemicals [15].

BBR, also known as BBR hydrochloride, is isolated from the rhizomes of the *C. chinensis* [16]. BBR has poor water solubility and is unstable, making it unsuitable for direct use as a drug. BBR hydrochloride is the hydrochloride salt of BBR, which is the hydrochloride form of isoquinoline alkaloids. BBR hydrochloride is easily soluble in water and stable and can be made into pills and capsules for easy consumption and absorption, exerting its pharmacological effects. There have been literature reports that BBR hydrochloride was used to study the effects of BBR [16–18]. Therefore, this study also used BBR hydrochloride intervention to investigate the HDIs.

Glipizide is a second-generation sulfonylurea antidiabetic drug. It is principally metabolized to inactive metabolites by genetically polymorphic CYP2C9 enzyme [5]. Meanwhile BBR could inhibit CYP2D6 in a quasi-irreversible manner, the combination of substances with inhibitory potential of P-gp and CYP (CYP2C19, 2D6, and 3A4) may increase the plasma exposure of BBR [19]. Therefore, the pharmacokinetic HDIs based on CYP450 may occur.

In Type II diabetes patients, the AUC_0-∞_ of glipizide increases two-fold after multiple dosage regimen in comparison with a single dose of glipizide. Furthermore, the *C*_max_ of glipizide increased in fasting conditions compared to the nonfasting state in diabetic individuals [20]. The way in which glipizide interacts with genetic variations may increase the risk of hypoglycemia. The combination of *Syzygium cumini* and glipizide may enhance the control of blood glucose and blood lipid in patients with Type 2 diabetes [21]. *Andrographis paniculata* (Burm. f.) ethanolic extract administration significantly improved the *C*_max_ and AUC_0−t_/AUC_0−∞_ of glipizide values in normal and diabetic rats, and Andrographolide significantly reduced the bioavailability of glipizide in diabetic rats with small values of T_1/2_, *C*_max_, and AUC_0−t_/AUC_0−∞_ [10].

After intervention with BBR, there were significant differences in the main pharmacokinetic parameters (*C*_max_, AUC_(0-t)_, AUC_(0-∞)_, and CL) of glipizide compared to when used alone. BBR can increase the plasma exposure of glipizide. This might lead to adverse reactions such as hypoglycemia while increasing the hypoglycemic effect.

With the intensification of global population aging, new diseases, adverse reactions, and the lack of effectiveness of many drugs, there will be more herbal and clinical drug combinations, as well as other treatment options. Strengthening effective research on the effectiveness and safety of herbal and other combination therapy options is a necessary approach for the scientific community to provide evidence of possible interactions, and increasing financial support for research on this plant–drug interaction is also an urgent task. Therefore, it is necessary to continue basic and clinical pharmacology research, as well as pharmacovigilance research and the regulatory patterns of this combination therapy [22]. For clinical doctors, it is important to guide them in understanding the herbal interactions caused by herbal medicines, herbal foods, and common plants. Patients can also be advised to avoid using conventional drugs, plant-based drugs, and foods with limited treatment windows [23].

## 5. Conclusions

A new UPLC-MS/MS method had been successfully implemented and applied for the determination of glipizide in beagle dog plasma. BBR could affect the pharmacokinetic parameters of glipizide in beagle dogs, thereby inhibiting its metabolism and increasing its plasma exposure. In clinical practice, attention should be paid to the possible occurrence of HDIs when BBR is used in combination with glipizide.

## Figures and Tables

**Table 1:** The precision and accuracy of glipizide in beagle dog plasma (*n* = 6).

Added (ng/mL)	Intraday		Interday		Matrix effect (%)	
	RSD (%)	Accuracy (%)	RSD (%)	Accuracy (%)	Mean ± SD	RSD (%)
10	5.96	99.23	7.93	98.74	98.20 ± 7.72	7.86
25	4.93	98.64	5.39	101.18	96.40 ± 4.73	4.91
500	2.29	98.76	4.43	102.77	102.86 ± 3.31	3.22
1500	1.89	102.43	2.78	99.25	102.52 ± 1.16	1.57

**Table 2:** Results of stability of glipizide under four different conditions (*n* = 6).

Added (ng/mL)	Autosampler 4°C, 6 h		Room temperature, 3 h		Three freeze-thaw		−20°C, 4 weeks	
	RSD (%)	Accuracy (%)	RSD (%)	Accuracy (%)	RSD (%)	Accuracy (%)	RSD (%)	Accuracy (%)
25	5.82	98.71	5.17	100.19	2.57	95.74	3.82	96.66
500	3.68	97.82	3.87	103.63	4.65	101.28	2.40	97.44
1500	1.92	101.80	1.65	97.65	1.19	102.25	1.74	98.49

**Table 3:** The main parameters of pharmacokinetics of glipizide in beagle dogs (*n* = 6, mean ± SD).

Parameters	Glipizide alone	Glipizide after BBR intervention
*T*_max_ (h)	3.50 ± 0.55	3.83 ± 1.17
*C*_max_ (ng/mL)	727.37 ± 244.89	1110.74 ± 249.67^∗^
t_1/2_ (h)	3.74 ± 0.81	4.12 ± 0.42
MRT_0⟶t_ (h)	7.18 ± 0.29	7.46 ± 0.48
MRT_0⟶∞_ (h)	7.69 ± 0.43	8.06 ± 0.56
CLz/F (L/h/kg)	0.13 ± 0.05	0.07 ± 0.02^∗^
Vz/F (L/kg)	0.71 ± 0.39	0.44 ± 0.13
AUC_0⟶t_ (ng/mL·h)	5867.62 ± 2296.64	9405.64 ± 2700.87^∗^
AUC_0⟶∞_ (ng/mL·h)	5966.88 ± 2310.90	9646.21 ± 2707.52^∗^

^∗^Compared with glipizide alone, the difference was statistically significant (*p* < 0.05).

## Data Availability

The data that support the findings of this study are available from the corresponding author upon reasonable request.
